# The Nature and Timing of Tele-Pseudoscopic Experiences

**DOI:** 10.1177/2041669515625793

**Published:** 2016-01-20

**Authors:** Stephen Palmisano, Harold Hill, Robert S Allison

**Affiliations:** Centre for Psychophysics, Psychophysiology and Psychopharmacology, School of Psychology, University of Wollongong, NSW, Australia; School of Psychology, University of Wollongong, NSW, Australia; Centre for Vision Research, York University, Ontario, Canada

**Keywords:** Stereopsis, pseudoscopic viewing, depth perception, space perception

## Abstract

Interchanging the left and right eye views of a scene (pseudoscopic viewing) has been reported to produce vivid stereoscopic effects under certain conditions. In two separate field studies, we examined the experiences of 124 observers (76 in Study 1 and 48 in Study 2) while pseudoscopically viewing a distant natural outdoor scene. We found large individual differences in both the nature and the timing of their pseudoscopic experiences. While some observers failed to notice anything unusual about the pseudoscopic scene, most experienced multiple pseudoscopic phenomena, including *apparent scene depth reversals*, *apparent object shape reversals*, *apparent size and flatness changes*, *apparent reversals of border ownership*, and even complex *illusory foreground surfaces*. When multiple effects were experienced, patterns of co-occurrence suggested possible causal relationships between *apparent scene depth reversals* and several other pseudoscopic phenomena. The latency for experiencing pseudoscopic phenomena was found to correlate significantly with observer visual acuity, but not stereoacuity, in both studies.

## Introduction

Our laterally separated eyes receive different perspective views of the same scene. One direct consequence of this horizontal eye arrangement is that the angular subtense between environmental objects separated in depth differs for the left and right eyes (in terms of angular magnitude and sign). Wheatstone (1838) showed that these horizontal binocular disparities can generate powerful impressions of depth, known as binocular stereopsis. Until quite recently, it was assumed by most that the benefits of stereopsis for depth perception were restricted to viewing, and interacting with, near objects and surfaces (e.g., Arsenault & Ware 2004; Cutting & Vishton, 1995; Gregory, 1966; Palmer, 1999). For example, Gregory (1966) claimed that we are “effectively one-eyed for distances greater than about twenty feet” (p. 53). However, stereopsis is now known to contribute to depth perception at considerably larger distances (see Allison, Gillam, & Vecellio, 2009; Gillam, Palmisano, & Govan, 2011; Palmisano, Gillam, Govan, Allison, & Harris, 2010).

In 2010, Palmisano et al. conducted an experiment inside a disused railway tunnel aimed at determining the limiting range of stereopsis. Pairs of LED targets were presented either in complete darkness or with the tunnel environment lit just as far as the nearest LED. Importantly, in all the conditions tested, the only information available about the depths between the two LED targets came from binocular disparity. Under these *reduced cue* conditions, we found that binocular (but not monocular) perceptions of depth magnitude increased with LED depths up to 248 m (the maximum LED depth separation that could be examined in this tunnel using an observation distance of 40 m to the nearest LED). However, as stereopsis was clearly operational at the largest distances and depths examined in this study, we still do not know its actual limiting range. Since good stereoscopic observers are able to detect depth separations corresponding to binocular disparities of only a few seconds of arc (Howard, 1919), the maximum useful range of stereopsis could in principle exceed 1 km (based on Allison et al.’s 2009 geometric analysis which conservatively estimated stereoacuity at ∼10 arcsec).

**Figure 1.**
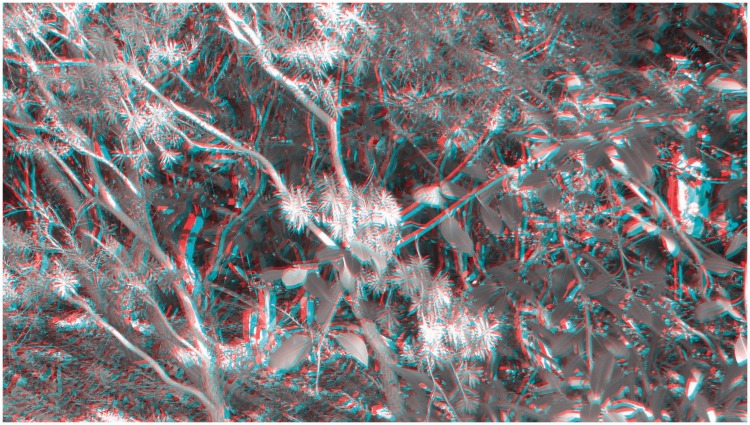
Red–cyan anaglyph stereo photograph presenting a rather cluttered natural scene. A normal scene can be observed with the red filter of the anaglyph glasses over the *left* eye. Switching the red filter to the *right* eye produces a reversed disparity scene. During this pseudoscopic viewing, the perception of globally consistent 3D layout can be difficult or even impossible for *reversing* observers. However, the pseudoscopic photograph is perceived as a normal 3D scene by nonreversers. This stereo photograph was taken with a Panasonic LUMIX 3D1 camera and converted into an anaglyph figure using StereoPhoto Maker (http://stereo.jpn.org/eng/stphmkr/).

Interestingly, more than a century ago, Stratton (1898) proposed that the pseudoscopic viewing of natural scenes could be used to determine the limiting range of stereopsis under *full cue* conditions (as opposed to the reduced cue conditions used in our 2010 tunnel experiment). A pseudoscope presents what is normally seen by the left eye to the right eye and vice versa (Wheatstone, 1852)—thereby putting binocular disparity in conflict with a variety of monocular sources of information about the spatial layout (reversing the sign of the available binocular disparities; so that uncrossed binocular disparities become crossed binocular disparities and vice versa). Not surprisingly, the pseudoscopic viewing of natural scenes can produce vivid stereoscopic effects (see Figure 1; Ewald & Gross, 1906; Kalaugher, 1987; Shimojo & Nakajima, 1981; Stratton, 1898; Wallin, 1905; Wheatstone, 1852).

According to Stratton (1898), the most striking of these pseudoscopic experiences is the apparent reversal of the scene’s depth order: “A tree, for example, between the person and a background of other trees may now seem to lie beyond those trees and to be seen through them” (p. 635). He argued that stereopsis continues to operate well beyond distances where such apparent scene depth reversals can be seen and proposed that the actual limiting range of stereopsis could only be identified by rapidly alternating between normal and pseudoscopic viewing. According to Stratton, the limiting range of stereopsis would correspond to the distance at which perceptible differences between normal and pseudoscopic viewing were lost. He argued that scenery beyond this distance, in the outermost zone, should look equally flat during both types of viewing (as opposed to appearing to have more depth during normal viewing). Based on his own observations using this technique, Stratton estimated that his limiting range of stereopsis was 580 m.

The current study was not directly aimed at testing Stratton’s ideas about the limits of stereopsis. Instead its primary purpose was to systematically examine the nature and the timing of pseudoscopic phenomena experienced when viewing natural outdoor scenes. Since the role that stereopsis plays at distances beyond personal (or interaction) space has historically received little examination, we were also particularly interested in the pseudoscopic phenomena generated by viewing distant scenes. This knowledge is necessary as a precursor for generally applying Stratton’s idea, as his technique for determining the limiting range of stereopsis assumes that the effects of pseudoscopic viewing are quickly perceived as well as robust and similar for individuals with normal binocular/stereoscopic vision (because the technique relies on the detection of differences between normal and pseudoscopic viewing during rapid view alternation).

Anecdotal reports suggest that there can be substantial differences in both the nature and timing of pseudoscopic experiences. For example, Wheatstone (1852) noted that “these appearances are not always immediately perceived” (p. 13) and “the transition from the normal to the converse perception is often gradual” (p. 15). Similarly, Kalaugher (1987) noted that “pseudoscopic effects … may take a long time to develop, if indeed they develop at all” (p. 372). Such reports also suggest that there are a variety of possible pseudoscopic experiences, some of which are more readily experienced than others. For example, it has also been claimed that the perceived depth order of the environment reverses more often than the perceived shape and curvature of individual objects (see Stratton, 1898; Wheatstone, 1852).

**Figure 2.**
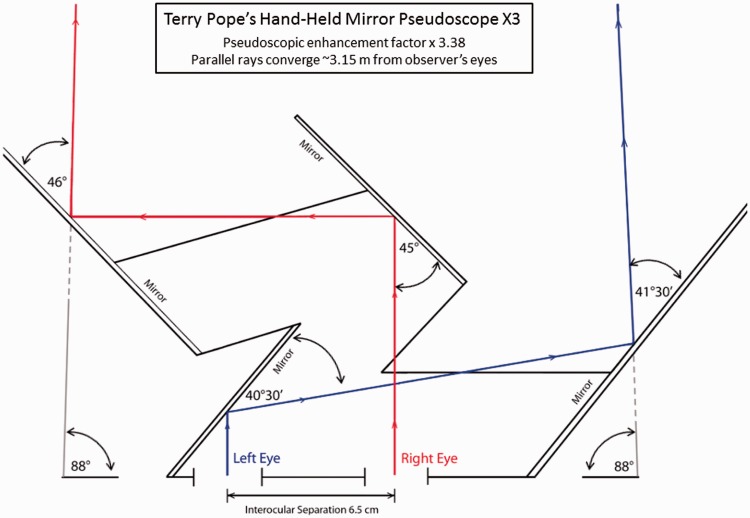
Ray diagram of Terry Pope’s hand-held Mirror Pseudoscope X3 (www.phantascope.co.uk; info@phantascope.co.uk). Effective interocular separation with this pseudoscope was 22 cm producing a pseudoscopic enhancement factor of 3.38 (assuming an observer interocular separation of 6.5 cm). Because the left and right eyes’ views were reversed, the relationship between vergence responses and physical distance was also reversed. The observer diverged his or her eyes in order to fixate objects closer than 3.15 m and converged them to fixate objects that were further away. Objects at infinity required a convergence angle of ∼4° whereas objects at a closer distance of 3.15 m required 0° convergence (parallel visual axes corresponding to fixation at infinity in normal viewing). In terms of the distance at which the pseudoscopic and equivalent telestereoscopic images should not *move* when switching from tele-pseudo to tele-stereo viewing, this point should lie between the near alignment point of 3.15 m and infinity (where the eyes are converged by ∼4°).

Pseudoscopic experiences are often difficult for observers to describe (particularly if they are not vision scientists or artists such as Terry Pope). This makes it challenging for experimenters to distinguish between naïve observers: (a) not experiencing these effects and (b) experiencing them but having difficulty describing them. Exaggerated pseudoscopic viewing has been repeatedly reported to produce more powerful and robust reversal effects (e.g., Kalaugher, 1987; Matthews, Hill, & Palmisano, 2011; Stratton, 1898). Unlike *normal* pseudoscopic viewing (where disparity magnitudes are maintained but disparity signs are reversed), disparity magnitudes are artificially increased during *exaggerated* pseudoscopic viewing (e.g., in the case of Kalaugher’s study he had a simulated interocular distance of 13 m). Since pseudoscopic experiences are reportedly accentuated by exaggerated pseudoscopic vision (Kalaugher, 1987; Pope, 2008; Stratton, 1898), we chose to use a commercial device with a pseudoscopic enhancement factor of 3.4^1^ in the current studies (see Figure 2). According to Terry Pope (the device designer), this particular pseudoscopic enhancement factor “makes it easy to have continuous pseudoscopic experiences, which as will be seen, are bizarre” (Pope, 2008, p. 7; see also Walker, 1986). His mirror pseudoscope also had two other potential advantages over most competing pseudoscopes (especially prismatic ones): (a) it had a relatively large field of view (it stimulated regions of the retina that were ∼90° wide in the left eye and ∼32° wide in the right eye; including a ∼28° wide region of binocular overlap), and (b) it did not result in any lateral inversion (Kalaugher, 1987).

## Field Study 1 (Pilot)

**Figure 3.**
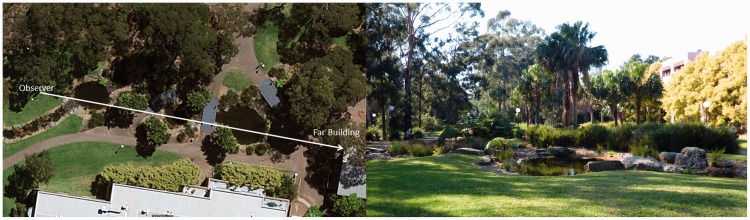
Bird’s eye view (left) and observer view (right) of the natural outdoor scene used for the pseudoscopic viewing experiment.

We examined the subjective experiences of 76 naïve binocular observers when viewing a natural outdoor scene through our pseudoscope. The natural scene consisted of a grass covered ground plane, a small pond surrounded by rocks, pole-mounted outdoor lights, trees and bushes at a variety of distances from the observer, as well as more distant buildings (see Figure 3). Accordingly, it provided a wide variety of monocular information about its 3D layout (both distance and depth cues). Field Study 1 examined the extent to which there are individual differences in the nature and the timing of the subjective experiences produced by tele-pseudoscopically viewing this particular scene. We also measured stereoacuity and basic visual acuity in order to see whether differences in these perceptual abilities were related to the likelihood and timing of tele-pseudoscopic experiences. As we were particularly interested in larger distances and real scenes, we chose a natural outdoor scene where the furthest visible objects (e.g., the far building) were approximately 75 m away (confirmed by Google Maps).

### Methods

#### Observers

In sum, 76 undergraduate students at the University of Wollongong (18 males and 58 females; mean age 25.3 years; age range 22–53 years) participated in this experiment for course credit. Observers with corrected vision always wore their normal glasses or contact lenses (i.e., during both the *vision testing* and *pseudoscopic viewing* phases of the study). While all of the observers had binocular vision, stereoacuities were found to range from 40 arcsec (or better) to 2,000 arcsec or worse (the upper limit of the Randot Butterfly Stereotest). Binocular visual acuities were found to range from 20/13 to 20/50. The Wollongong University Ethics Committee approved the study in advance (HE12/311), and each observer provided verbal informed consent before participating. These protocols were in accordance with the ethical standards laid down in the 1964 Declaration of Helsinki.

#### Apparatus

Stereoacuity was measured using the Random Dot Stereo Butterfly test (from Stereo Optical Co., Inc; http://www.stereooptical.com/shop/stereotests/butterfly-stereotest/) at the recommended testing distance of 16 in. (∼41 cm). Visual acuity was measured using a Snellen Eye Chart, viewed at the recommended testing distance of 20 feet (∼6 m). A mirror X3 pseudoscope (Phantascope; http://www.phantascope.co.uk/pages/about_pesu_handheld.html) was used for the pseudoscopic viewing of the natural outdoor campus scene. Finally, the observers’ pseudoscopic effect latencies were self-timed via a stopwatch.

#### Procedure

Pseudoscopic viewing occurred at a single location on the University of Wollongong’s main campus (around midday on sunny, not overcast, days). Figure 3 (a photograph taken from the observer’s actual viewing position) shows the scenery visible at the testing site. Each observer was taken to this testing location and asked to describe what they saw as they looked through the pseudoscope. Observers were to report anything unusual or interesting during their first 5-minute pseudoscopic exposure to the scene and on the basis of this information they were classified as either being *reversers* or *nonreversers*. After a 5-minute long period of normal binocular viewing of this scene (i.e., natural disparity viewing), they then pseudoscopically viewed the same scene for a second time. Pseudoscopic effect latency was measured on this second pseudoscopic exposure—observers started the stopwatch when the pseudoscopic viewing period began and stopped it when they first noticed something *strange/unusual*. They then again described their experiences to the experimenter. Only reported strange/unusual experiences that could potentially be pseudoscopic in origin were counted (if the description of these experiences did not meet this criterion, then the timing data was discarded and the timing trial was repeated).^2^ After the pseudoscopic viewing phase of the experiment, observers were escorted to an indoor laboratory, where their stereoacuity and binocular visual acuity were measured under controlled conditions.

### Results

We found large individual differences in the subjective experiences of pseudoscopic viewing. Based on spontaneous descriptions during the two periods of pseudoscopic viewing, we identified five distinct types of strange/unusual experiences: (a) *Apparent scene depth reversals*, where physically near objects and surfaces were misperceived to be far and vice versa (e.g., “the tree on the left appeared to be far away before [normal viewing], but now appears nearer to me than the one on the right”). While such depth reversals could occur across the entire scene, they were often reported as patchwork effects (i.e., most salient in terms of the relations between a subset of environmental objects/surfaces). (b) *Apparent object shape reversals*, where convex objects were misperceived as concave and vice versa (e.g., “the trunk of that palm tree looks like it is hollowed out”); (c) *Apparent size and flatness changes*, where physically far objects were misperceived to be smaller and flatter during pseudoscopic (compared to normal) viewing (e.g., “trees and the other objects in the scene now look unnaturally flat”); (d) *Apparent reversals of border ownership*, where the edges of physically far objects were misperceived to belong to nearer parts of the scene and the outlines of physically near objects could be seen as belonging to the background (e.g., “The edge formed where the leaves of the two trees overlap looks weird … back-to-front”); and (e) *Complex illusory surfaces*,^3^ where new and distinct foreground surfaces were perceived during pseudoscopic viewing. Such pseudoscopic experiences appeared to be more than simply *depth reversals*, as observers were aware that such surfaces did not exist in reality and while they were textured with real scene detail, other parts of these surfaces could be entirely illusory (e.g., “It is blowing my mind because I know there can’t really be a surface there”; “That region looks like a cardboard cut-out”; “It feels like I am looking at that part of the scene through stained glass”). Although previous research has shown that pseudoscopic viewing can alter perceptions of surface color or shading for simplified scenes (e.g., Bloj, Kersten, & Hurlbert, 1999; Logvinenko & Menshikova, 1994), we had no spontaneous reports of such percepts when viewing our complex natural scene (possibly because they were less salient in this situation compared to the phenomena described earlier).

**Figure 4.**
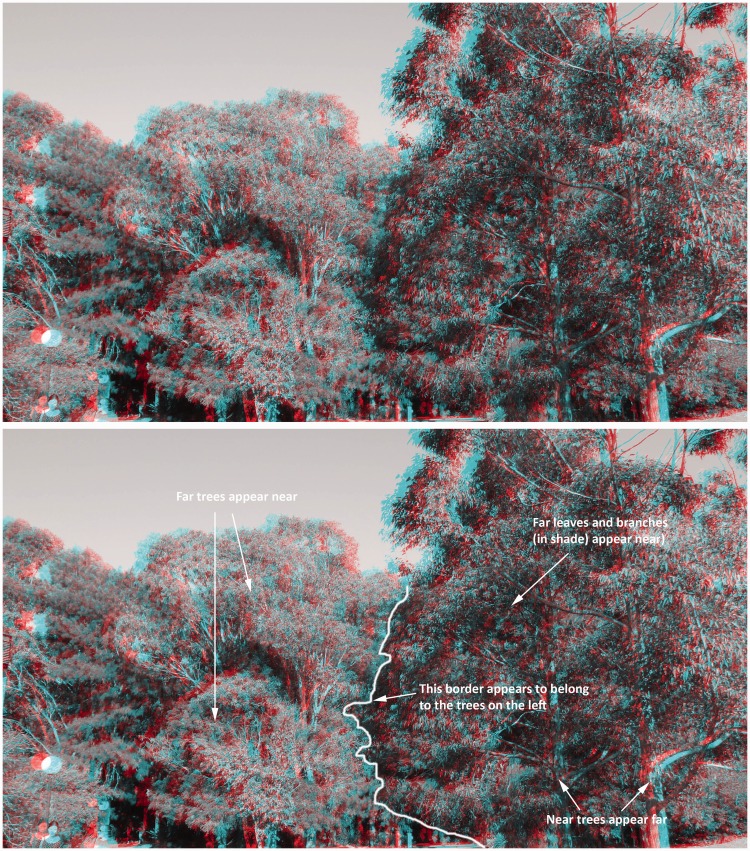
(Top) Example of *apparent scene depth reversals* and a *reversal of border ownership* (red–cyan anaglyph photograph—red filter again over the *right* eye for pseudoscopic viewing). (Bottom) Typical subjective experiences are overlaid on this same photo, including an *apparent reversal in border ownership* between the trees on the left and the right sides of this scene. The original stereo photographs were taken with a Panasonic LUMIX 3D1 camera and then converted into anaglyph figures using StereoPhoto Maker.

**Figure 5.**
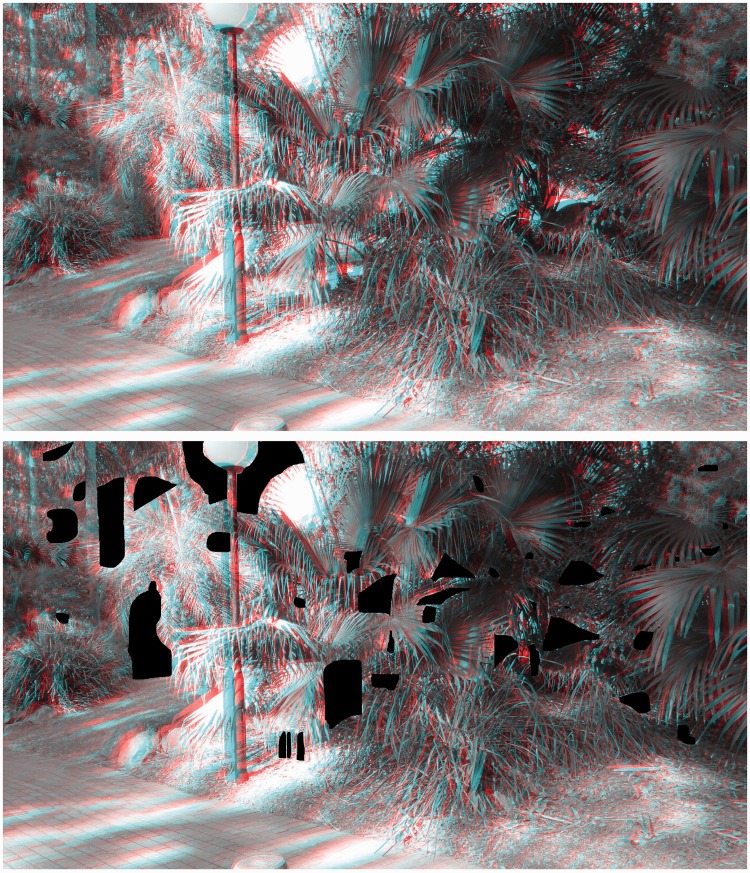
(Top) Examples of *complex illusory surfaces* (red–cyan anaglyph photograph—red filter over the *right* eye for pseudoscopic viewing). (Bottom) Locations of some salient apparent surfaces seen by SP in this particular scene. The original stereo photographs were taken with a Panasonic LUMIX 3D1 camera and converted into anaglyph figures using StereoPhoto Maker.

Figures 4 and 5 provide natural disparity stereoscopic photographs of two parts of this testing scene. Figure 4 attempts to illustrate *apparent scene depth reversals* and *apparent reversals of border ownership*, whereas Figure 5 attempts to illustrate *complex illusory surfaces*, produced by pseudoscopic viewing. Given large differences in the timing of reported pseudoscopic effects between individuals, we also analyzed the time take to report the first pseudoscopic experience. Pseudoscopic effects were almost instantaneous for some, delayed and difficult to see for others, and impossible to see for the rest. For some reversers, pseudoscopic experiences were very robust—once obtained they persisted for almost the entire pseudoscopic viewing period. However, pseudoscopic effects could also be transient or bistable for other reversers. Pseudoscopic effects often evolved over the course of the trial. For example, some observers, who later experienced *apparent scene depth reversals*, initially reported that objects (such as trees or the lamp posts) which they knew to be at different physical distances appeared to be equally far away. In terms of the onset latencies of these pseudoscopic experiences, such preliminary symptoms (e.g., this perceptual alignment of physically depth separated objects) were deemed to be sufficient for a valid pseudoscopic timing response.

**Figure 6.**
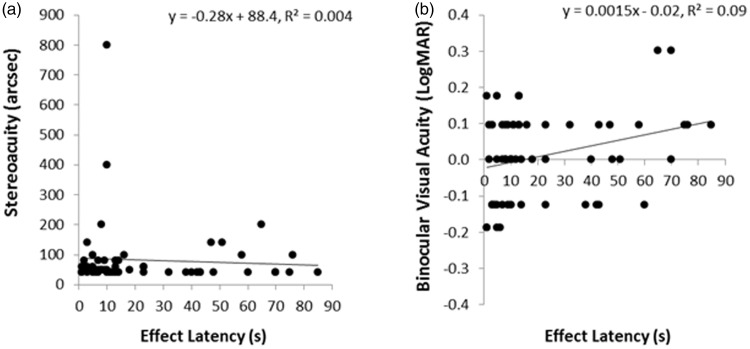
Relationship between pseudoscopic effect latency and (a) stereoacuity and (b) binocular visual acuity. LogMAR = Log minimum angle of visual resolution (20/20 corresponds to a logMAR score of 0; positive/negative scores are worse/better than 20/20).

We next investigated whether these pseudoscopic effect latencies could be predicted by either the observer’s general or stereospecific visual abilities. Nineteen of our 76 binocular observers did not appear to experience any pseudoscopic effects—they reported that the test scene looked perfectly normal to them even though all of the available test scene disparities were reversed and exaggerated. Only six of these nonreversers were effectively static stereoblind (they had stereoacuities worse than 2,000 arcsec according to the Random Dot Butterfly Test). The remaining 13 had functional static stereovision—all had stereoacuities better than 800 arcsec (mean stereoacuity was 99.7 arcsec; standard deviation was 158 arcsec). Thus, we were unable to identify all of our nonreversers based on static stereoability alone. These 19 nonreversers all had binocular visual acuities of 20/50 or better (13 had 20/20 or better vision, and only 6 had vision that was worse than 20/20). We then examined whether stereoacuity or binocular visual acuity could predict the pseudoscopic effect latencies of our reversers. While pseudoscopic effect latency was found to be independent of stereoacuity, *R*^2 ^= 0.003, *F*(1, 56) = −0.19, *p* = .66 (see Figure 6(a)), we did find a relationship between pseudoscopic effect latency and binocular visual acuity. Pseudoscopic effect latencies were significantly reduced for observers with superior binocular visual acuities, *R*^2 ^= 0.09, *F*(1, 56) = 5.40, *p* = .02 (see Figure 6(b)). Due to the presence of a large number of *ties*, we subsequently confirmed this relationship with Spearman (*r*(55) = .30, *p* = .02) and Tau-b (*r*(55) = .24, *p* = .02) correlations.^4^

### Discussion

This exploratory field study examined the pseudoscopic experiences of 76 individuals when viewing a distant natural outdoor scene. Their spontaneous descriptions were subsequently categorized into five distinct types of pseudoscopic experiences: *apparent scene depth reversals*, *apparent shape reversals*, *apparent size and flatness changes*, *apparent reversals of border ownership*, and *complex illusory surfaces.* While these different pseudoscopic experiences often appeared to co-occur, the current study did not provide systematic data on this issue. Accordingly, Field Study 2 was designed to provide empirical data on the frequency and co-occurrence of these different phenomena.

In the current study, the experimenters deliberately did not provide observers with any information about the possible strange/unusual symptoms they might experience during pseudoscopic viewing (they only diagnosed spontaneous reports of their experiences). Thus, it is possible that the large number of observers classified as nonreversers in this study (i.e., 19 out of the total 76 observers) might reflect an overestimate due the conservative methodology used. Field Study 2 was also designed to investigate this possibility. Instead of spontaneous responses, this follow-up study used careful and systematic questioning to determine the occurrence of any/all of the pseudoscopic phenomena identified by the pilot study.

Field Study 1 was also aimed at determining how long these pseudoscopic experiences took to develop. We found that pseudoscopic effects were almost instantaneous for some, delayed and difficult to see for others, and impossible to see for the remainder. In terms of explaining these individual differences, stereoacuity was not found to distinguish reversers from nonreversers, nor was it found to significantly predict pseudoscopic effect latency. However, binocular visual acuity was found to significantly predict individual differences in pseudoscopic effect latency—although this relationship only accounted for approximately 10% of the variance. Field Study 2 was also run to test whether this relationship could be replicated.

## Field Study 2

Field Study 2 examined the frequencies of experiencing each of the five different pseudoscopic phenomena identified in Field Study 1. These pseudoscopic experiences and their latencies were recorded for a completely different group of 48 naïve observers. In this study, we also measured *monocular visual acuities* for the left and right eyes separately, as well as *stereoacuity*, in order to reexamine the relationships of these measures to pseudoscopic effect latencies.

### Method

#### Observers

A total of 48 undergraduate students at the University of Wollongong (18 males and 30 females; mean age 24.3 years; age range 21–45 years) participated in this experiment for course credit. While all of these observers had binocular vision, their stereoacuity ranged from 40 arcsec (or better) to 2,000 arcsec. Their monocular visual acuities (again measured at 6 m, but this time assessed for the left and right eyes separately) ranged from 20/10 to 20/70. None of these observers had participated in Field Study 1 and they had not experienced pseudoscopic viewing before.

#### Procedure

In Field Study 2, the monocular visual acuities of each observer’s left and right eyes, as well as their stereoacuity, were measured by one experimenter under controlled laboratory conditions. Observers then walked across campus to a second experimenter (who was completely unaware of their performance during vision testing) located at the pseudoscopic testing site used in Field Study 1. This second experimenter then asked observers to freely look through the pseudoscope for 3 minutes and report if they saw anything strange or unusual. After a 5-minute break of normal binocular viewing, observers were given their second pseudoscopic exposure to the scene. During this second pseudoscopic viewing period, observers were instructed to look directly ahead (i.e., over the pond and towards the trees and buildings in the distance—see Figure 3) and their pseudoscopic effect latency was timed. Each observer started their stopwatch when pseudoscopic viewing began and stopped it when they experienced their first unusual/strange pseudoscopic effect. These experiences were described to the experimenter in detail and categorized into the five subjective experiences identified earlier in Field Study 1 (i.e., *apparent scene depth reversals*, *apparent shape reversals*, *apparent size and flatness changes*, *apparent reversals in border ownership*, and *complex illusory surfaces*) by careful questioning.

During this *experience categorization* phase of the experiment, the experimenter went through the following checklist:

- “Please direct your attention to the two trees directly ahead of you, which tree looks nearer? The one on the left or the one on the right?”
- “Do any of the familiar objects in the scene appear to be inside out? For example, concave as opposed to convex (or vice versa)? That is, hollow as opposed to bulging (or vice versa)?”
- “Do any familiar objects or features of the scene look unusual to you in terms of either their size (larger/smaller) or shape (e.g., thinner/fatter)? If so, which features and how do they appear?”
- “Please direct your attention to the leafy parts of the overlapping trees ahead of you, and in particular, to the border formed between them. Which of these trees does this border appear to belong to? The one on the left or the one on the right?”
- “Please direct your attention to the space between the trunks of these two trees. Can you describe to me how this region of the scene appears to you?” (If the observer reported symptoms consistent with the existence of a *complex illusory surface*, he/she was then asked to report the locations of at least one other such surface in the scene for confirmation).

### Results

Five of our 48 binocular observers did not report any pseudoscopic effects. They did not think that anything looked unusual or out-of-the-ordinary (even though all of the available scene disparities were reversed and exaggerated). These five observers were thus classified as nonreversers. Only one of them appeared to be static stereoblind (she could not see the stereo butterfly and her stereoacuity was worse than 2,000 arcsec according to the Random Dot Butterfly Test; she also had monocular visual acuities of 20/30 and 20/70 in her two eyes). One other nonreverser had a stereoacuity of 400 arcsec. However, the remaining three nonreversers all had stereoacuities better than 40 arcsec. Again, stereo ability alone was not sufficient to identify all nonreversers. The five nonreversers had the following left and right eye visual acuities: 20/20 & 20/20; 20/25 & 20/25; 25/20 & 20/20; 25/20 &20/20; and 20/70 & 20/30.

**Figure 7.**
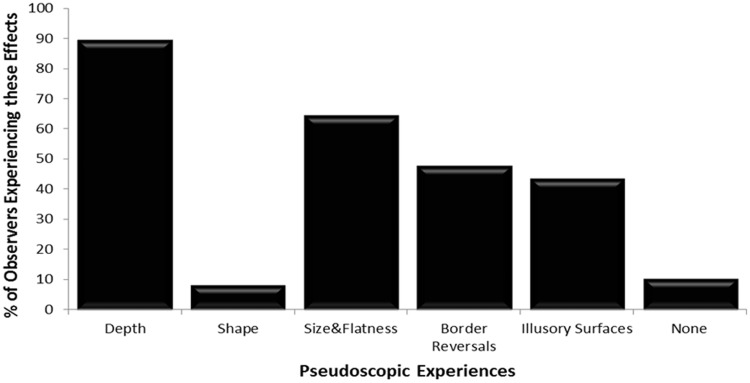
Percentages of observers (*n* = 48) who experienced each of the different pseudoscopic effects (*scene depth reversals*, *object shape reversals*, *size and flatness changes*, *reversals in border ownership* and *complex illusory surfaces*). The percentage of nonreversers is also shown (*none*).

Field Study 2 examined the likelihood of experiencing the different pseudoscopic phenomena previously identified by Field Study 1 (see Figure 7). Of the five pseudoscopic experiences identified, *scene depth reversals* were the most common (experienced by 90% of the 48 observers; i.e., all of the reversers), and *shape reversals* were the least common (8% of the 48 observers). *Size and flatness changes* (65% of the 48 observers^5^), *reversals in border ownership* (48% of the 48 observers), and *complex illusory surfaces* (44% of the 48 observers) were all quite commonly experienced. These five pseudoscopic phenomena were not mutually exclusive. Two or more of these phenomena were typically experienced simultaneously by reversers. While it was possible for all of them to be experienced in the same session (3 of the 43 reversers reported all five phenomena in the same session), it was more common for a subset of experiences to co-occur (13/43 reversers each experienced four of the phenomena, 7/43 experienced three of the phenomena, and 14/43 experienced two of the phenomena).

**Figure 8.**
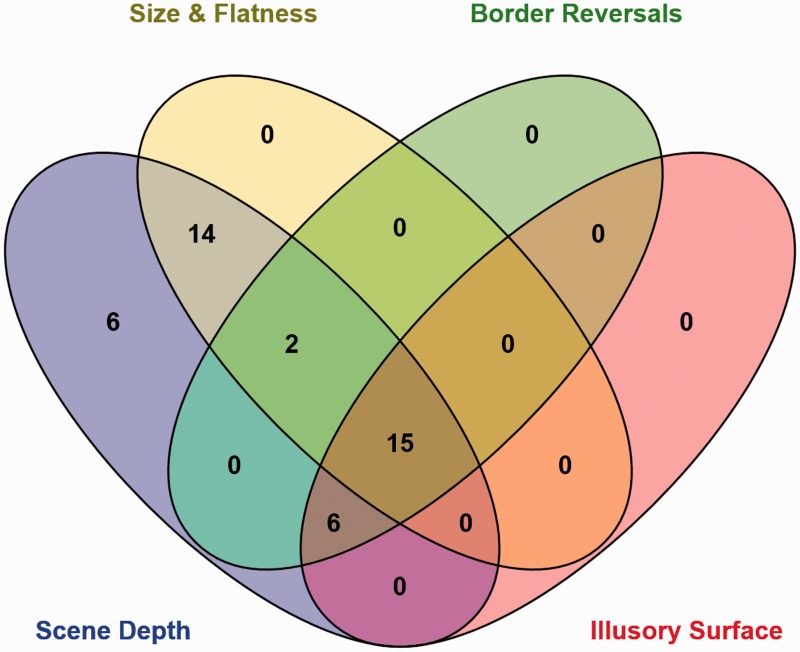
Venn diagram showing the relationships between *scene depth reversals*, *size and flatness changes*, *reversals in border ownership*, and *complex illusory surfaces* (*object shape reversals* are not shown as they were only experienced by four observers). The numbers of observers experiencing these phenomena, either in isolation or in combination, are provided.

When multiple pseudoscopic effects were experienced, their patterns of co-occurrence appeared consistent with *apparent scene depth reversals* being necessary for the perception of several of the other pseudoscopic phenomena (*size and flatness changes*, *reversals in border ownership*, and *complex illusory surfaces)*. As can be seen in the Venn diagram provided in Figure 8, (a) *size and flatness changes* only occurred when the observer also experienced *scene depth reversals*, (b) all reported cases of *reversals in border ownership* were accompanied by *scene depth reversals*, and (c) all reported cases of *complex illusory surfaces* were accompanied by *scene depth reversals*. Interestingly, Figure 8 shows that in all but 2 of the 23 cases, *reversals in border ownership* were also accompanied by *complex illusory surfaces—*suggesting that both *apparent scene depth reversals* and *apparent reversals in border ownership* aided in the perception of *complex illusory surfaces*.

**Figure 9.**
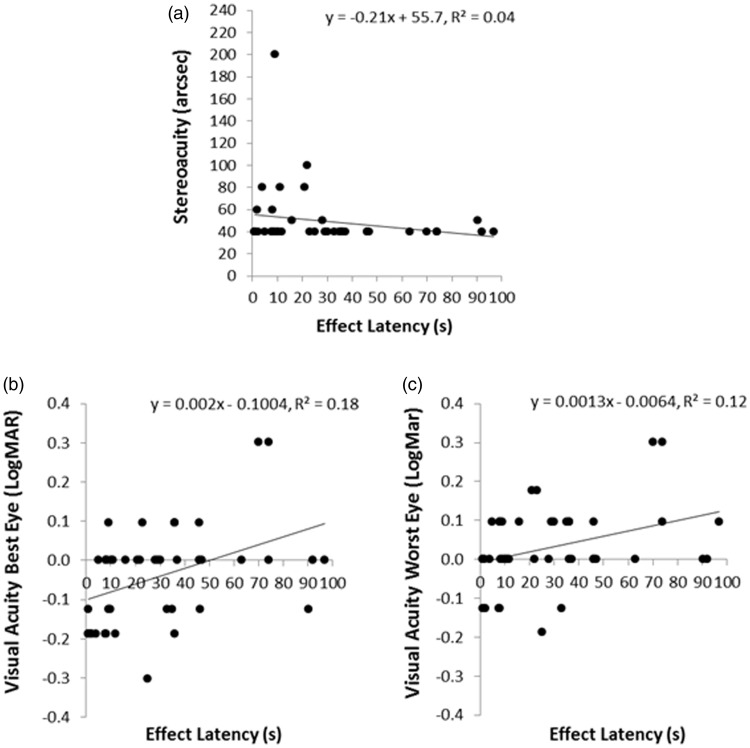
Relationships between pseudoscopic effect latency and (a) stereoacuity, (b) visual acuity (best eye), and (c) visual acuity (worst eye).

Next we looked at the time taken to experience these pseudoscopic effects. As in Field Study 1, only reversers were examined. This group of observers all had functional static stereovision and visual acuities no worse than 20/40 in each eye. We again found large individual differences in the time taken for reversers to experience pseudoscopic phenomena. These individual differences in pseudoscopic effect latency could not be explained by differences in stereoacuity, *R*^2 ^= 0.04, *F*(1, 42) = 1.83, *p* = .183 (see Figure 9(a)).

As in Field Study 1, pseudoscopic effect latencies were again significantly reduced for observers with higher visual acuities (i.e., smaller or negative LogMAR scores—see Figure 9(b) and (c)). While Field Study 1 only measured binocular visual acuity, Field Study 2 measured the monocular visual acuities of each of the observer’s eyes. The reversers we examined all had monocular visual acuities which ranged from 20/10 (i.e., −0.3 LogMAR) to 20/40 (i.e., +0.3 LogMAR). The visual acuity of their better eye was the best predictor of pseudoscopic effect latency—accounting for 18% of the variance (*R*^2 ^= 0.18, *F*(1, 42) = 9.13, *p* = .004). Nonparametric correlation based checks confirmed this relationship—reducing the likelihood that this finding was due to outliers (Spearman’s *r*(41) = .48, *p* = .001; Tau-B *r*(41) = .37, *p* = .002). While the visual acuity of the worse eye also significantly predicted pseudoscopic effect latencies (*R*^2 ^= 0.12, *F*(1, 42) = 5.72, *p* = .02), it should be noted that the acuities of the two eyes were highly correlated (Spearman’s *r*(41) = .76, *p* = .0001; Tau-B *r*(42) = .70, *p* = .0001). A final regression revealed that differences between the visual acuities of the two eyes did not significantly predict pseudoscopic effect latencies (*R*^2 ^= 0.07, *F*(1, 42) = 3.00, *p* = .091).

## General Discussion

As Wheatstone (1852) noted: “with the pseudoscope we have a glance, as it were, into another visible world, in which external objects and our internal perceptions have no longer their habitual relation with each other” (p. 12). Since the original invention of pseudoscopy by Wheatstone, these often vivid and bizarre pseudoscopic experiences have intrigued and fascinated perceptual researchers. Thus, it can be surprising for those of us who have experienced these phenomena so powerfully to read statements that “monoscopic cues … usually dominate the stereoscopic cues” during pseudoscopic viewing (e.g., Devernay & Beardsley, 2010, p. 11). Similarly, Shimojo and Nakajima (1981) also report that “when veridical monocular cues or familiarity cues were also presented normal depth would be experienced in spite of the reversal of disparity” (p. 392). Of course, the strength of these pseudoscopic effects are thought to depend in a large part on the viewing method used—with weaker or null effects being more common with pseudoscopes that have small fields of view and that result in lateral inversion (Kalaugher, 1987). However, pseudoscopic effects have also been anecdotally reported to be weak or absent under seemingly ideal viewing conditions (i.e., with large fields of view, all scene disparities reversed, and no lateral inversion). For example, accidental pseudostereopsis was apparently quite common when viewing 3D movies at the theatre in the past, either because the filters in front of the eyes, or the projectors, or the film reels themselves, were mixed up (Zone, 2005). Apparently the audiences in these situations often did not notice the reversed disparities and still thought they were seeing a 3D movie (albeit an uncomfortable or uninspiring one—Devernay & Beardsley, 2010; Zone, 2005).

Our field research has shown that perceptual experiences during tele-pseudoscopic viewing are far more complicated than the simple dominance of either *monocular cues* over *stereoscopic cues* or of *stereoscopic cues* over *monocular cues*. These tele-pseudoscopic experiences also appear inconsistent with a simple averaging of these stereoscopic and monocular cues. When all of the available scene disparities were reversed, there were large individual differences in both the nature and the timing of the resulting pseudoscopic experiences. The current findings demonstrate that some observers (nonreversers) failed to notice anything unusual about the pseudoscopically viewed scene (the scene looked normal and natural to them even with prolonged viewing). However, under identical conditions, most observers (reversers) experienced a variety of pseudoscopic phenomena. For these reversers, the reversed disparity information clearly competed with, and in some cases, powerfully overrode the available monocular information (such as occlusion—arguably the strongest ordinal monocular depth cue), the result being vivid pseudoscopic effects (either experienced almost instantaneously or developing gradually over time).

**Figure 12.**
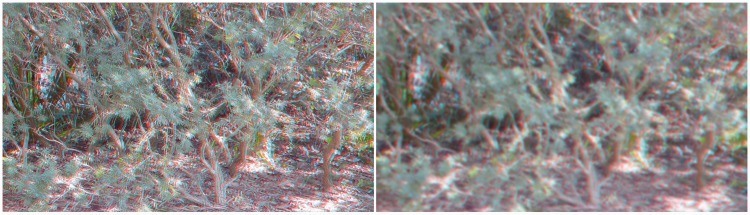
Original (left) and artificially blurred (right: Photoshop Gaussian blur with a radius of 8.5 pixels) red–cyan anaglyph pseudoscopic photographs. Pseudoscopic effects are still perceptible in the highly blurred anaglyph on the right (red filter over the *right* eye for viewing both pseudoscopic photographs).

It is possible that these intriguing individual differences in viewing experience were side effects of the Pope pseudoscope either (a) exaggerating the effective interocular distance (and consequently the scene disparities) or (b) altering the direction and magnitude of the vergence eye movements required to fixate different parts of the scene (see the caption of Figure 2 for a description). In terms of possibility (a), we note that the five different categories of pseudoscopic phenomena (discussed in Types of Pseudoscopic Experience section below) can still be seen when viewing Figures 1, 4, 5, and 12. As these stereophotographs were taken with a Panasonic LUMIX 3D1 camera that had only a 3-cm interaxial lens separation (i.e., less than the average interocular separation and considerably less than the 22 cm effective interocular separation of the Pope pseudoscope), this suggests that such pseudoscopic experiences also occur with normal/reduced-from-normal scene disparities. In terms of possibility (b), we note that the reversed direction of vergence eye movements is a by-product of any pseudoscopic viewing situation (since, by definition, the disparities are reversed in sign). However, we acknowledge that the magnitude of the vergence eye movements required and *mirror* point will vary (e.g., in a display based pseudoscope the mirror point would normally be the screen distance). We also acknowledge that it is currently unclear as to whether individual differences in pseudoscopic effect latency (discussed in Individual Differences in Pseudoscopic Effect Latency section below) are restricted to the Pope pseudoscope or generalize to other pseudoscopic viewing conditions.

### Types of Pseudoscopic Experience

In the present study, we identified five different categories of phenomena that could be experienced when pseudoscopically viewing our natural scene. These were not mutually exclusive, and in fact, several of these effects co-occurred for most observers. The most striking and unusual of these effects were the *complex illusory surfaces*, which were surprisingly common (44% of observers in Field Study 2) and are discussed in detail in the next section. Consistent with previous anecdotal reports, *apparent scene depth reversals* were the most commonly experienced of these pseudoscopic effects—reported by 90% of the observers in Field Study 2 (i.e., all of the reversers). In most cases, these scene depth reversals were straightforward for our naïve observers to see and describe (physically near objects in the scene were spontaneously reported to instead appear to be far away and vice versa). *Reversals in border ownership* were also quite common (reported by 48% of observers in Field Study 2) and easy to notice when attending to scene regions that contained near-far occlusions. However, Field Study 1 revealed that these border reversal effects could be difficult for naïve observers to spontaneously describe. Accordingly, carefully designed questions were used to identify border reversal effects in Field Study 2. *Apparent size and flatness changes* were also common (reported by 65% of observers in Field Study 2). However, observer reports suggested these effects were less salient than scene depth reversals and border reversals (they did not *jump out* in the same way). Objects that were further away (but appeared closer) merely appeared somewhat smaller and flatter.^6^ By contrast, consistent with Wheatstone’s (1852) own observations,^7^ *apparent object shape reversals* were found to be quite rare (reported by only 8% of observers in Field Study 2). However, these object shape reversals were quite striking whenever they were actually seen (convex objects, such as tree trunks, were reported to appear *hollowed out* or *inside out*). Interestingly, all of the observers that reported these *apparent object shape reversals* had stereoacuities better than 40 arcsec. These individuals may have been better able to utilize the fine relative disparities/disparity gradients required to process object shape (such as the small variations in disparity along a surface). While vertical disparity should not have affected the predicted depth order during pseudoscopic viewing, it is also possible that reversed patterns of vertical disparity might have affected *apparent shape reversals* and possibly the tolerance to pseudoscopic reversals by calibrating the available (reversed) horizontal binocular disparities (see Gillam, Chambers, & Lawergren, 1988; Rogers & Bradshaw, 1993).

As noted earlier, *apparent scene depth reversals* were the most commonly reported pseudoscopic experience. Observer reports strongly suggested that the experience of several other pseudoscopic effects logically depended on these changes in apparent scene depth/distance. Apparent *changes in size and flatness* as well as apparent changes in *border ownership* only occurred when there were also apparent depth reversals in the scene layout. Similarly, all reported cases of *complex illusory surfaces* were also accompanied by scene depth reversals. Furthermore all but 2 of the 23 cases of *reversals in border ownership* were also accompanied by complex illusory surfaces. Taken together, these observations suggested that (a) it was not possible to experience *reversals in border ownership* or *complex illusory surfaces* without experiencing apparent *scene depth reversals*, and (b) *reversals in border ownership* might also be important for generating the *complex illusory surfaces*.

#### Appearance and generation of the complex illusory surfaces

Of all the pseudoscopic reversal phenomena described earlier, the *complex illusory surfaces* were reported to be particularly *striking and unusual* by those who experienced them (often referred to as *bizarre* or *trippy*). These completely new (and in reality nonexistent) perceived surfaces could only be seen during pseudoscopic viewing. While they always appeared as foreground occluding surfaces, they were typically more than simply depth reversals (although the two pseudoscopic effects were clearly related). These complex illusory surfaces were formed by objects and surfaces that were actually present in the scene. However, they were perceptually reorganized in unusual/unfamiliar ways. Our own observations and observer reports (e.g., *ethereal surfaces* and *looking through stained glass*) revealed that these new surfaces (a) were often textured by a mixture of empty space and scene details from physically far regions and (b) were each bounded by perceptually continuous contour consisting of a variety of differing/nonmatching components/segments (i.e., patterns of luminance and color change across each perceived bounding contour often varied dramatically along its length).

**Figure 10.**
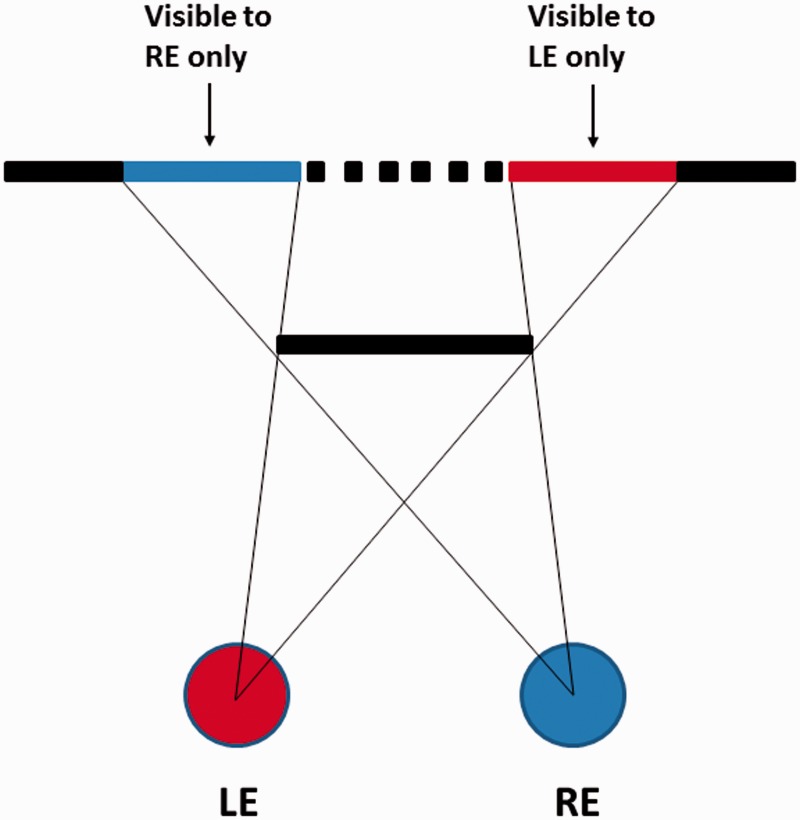
Bird’s eye view of an occluding surface with two *da Vinci* type monocular regions generated on the surface behind (LE = left eye; RE = right eye).

**Figure 11.**
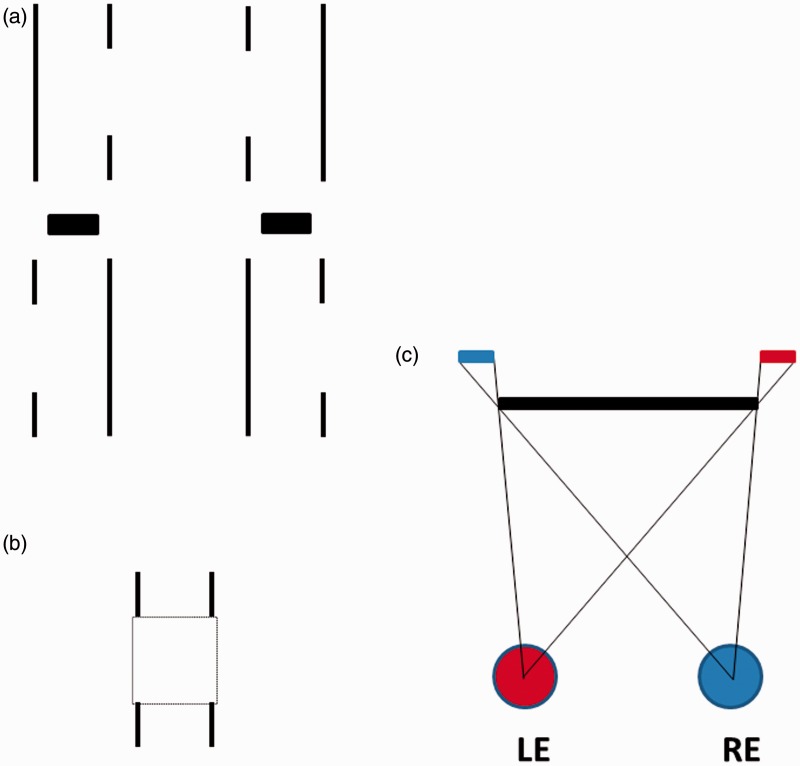
(a) Uncrossed fusion of the upper pair (or crossed fusion of the lower pair) of this stereogram generates a phantom rectangle, which appears to account for the monocular regions in the centers of the vertical lines. (b) The percept produced by free fusing the stereogram. (c) Gillam and Nakayama (1999) found that, as predicted from the geometry of this situation, the perceived depth of the phantom occluder increases with the thickness of the vertical stimulus lines.

Purely illusory foreground surfaces (i.e., phantom surfaces) have been previously reported in the literature under conditions in which monocular regions are generated in binocular images (e.g., Nakayama and Shimojo, 1990). Leonardo da Vinci noticed that such monocular regions occur in normal binocular vision whenever a nearer surface occludes a further textured surface (Richter, 1977; Wade, Ono, & Lillakas, 2001). For example, in the particular situation depicted in Figure 10, the left eye sees more of the background surface on the left, and the right eye sees more of the background surface on the right, generating two different but *valid* monocular regions. These monocular regions are deemed to be valid because they can be accounted for by the presence of the nearer binocularly visible occluder. However, Nakayama and Shimojo (1990) demonstrated that *phantom surfaces* (i.e., invisible foreground occluders) are perceived when *invalid* monocular regions occur in the binocular image. Gillam and Nakayama (1999) measured the perceived depth of such phantom surfaces under conditions similar to those depicted in Figure 11(a) (perceptual appearance and geometry are shown in Figure 11(b) and (c)). They found that the positions and depths of these phantom surfaces appeared to account for the absence of the expected feature in the other eye’s image. Something similar may have been happening here during the pseudoscopic viewing of our natural outdoor scene—even though *phantom surfaces* are traditionally thought of as being completely invisible foreground occluders (e.g., Harris & Wilcox, 2009), whereas our *complex illusory foreground surfaces* were a mixture of both empty space and real scene texture.

Monocular regions that are valid (i.e., can be accounted for by the presence of a nearer physical occluder) during the normal viewing of a scene should become invalid during pseudoscopic viewing of the same scene (at least, when perceived depths in that part of the scene are reversed). Thus, it is possible that *complex illusory occluding surfaces* were perceived during pseudoscopic viewing in our studies to account for the generation of invalid monocular regions in the natural outdoor scene. The purely illusory patches in these surfaces (possibly phantoms) appeared to contribute to their particularly strange/unusual (e.g., ethereal) overall appearance.

Previously, Häkkinen and Nyman (2001) showed using schematic stimulus displays, that *phantom surfaces*^8^ can capture ambiguous wall paper texture lying within them (all of the texture inside the region appeared to lie at the same depth as the bounding contours of the phantom surface). Interestingly, similar capture of natural scene texture appeared to be occurring with the *complex illusory surfaces* seen in our two field studies. While their internal surface texture was generated by objects lying at a variety of depths, the *complex illusory surfaces* themselves were usually reported as being irregularly shaped flat frontal planes lying in front of physically nearer scene regions. Of course, an alternative explanation could be that these illusory surfaces just appeared planar (if a distant scene was misperceived to be nearer then all of the disparity-based depth in the perceived foreground should have been compressed).

### Individual Differences in Pseudoscopic Effect Latency

As well as differences in the nature of the pseudoscopic effects experienced, we also found large individual differences in the time taken to experience these effects. What factors might be responsible for these differences in pseudoscopic effect latency? They were unlikely to have been due to binocular rivalry. Even though the observer’s left and right eye views were switched, and scene disparities were exaggerated by a factor of ∼3.4, the two eyes’ views remained remarkably similar (with the exception of monocular regions). Also, once pseudoscopic effects were fully developed they tended to be quite stable (not transient or bistable)—from that point on they were generally perceived continuously until the pseudoscopic viewing period ended.

We also examined whether these individual differences in pseudoscopic effect latency could be explained by either the observer’s general or stereospecific visual abilities. While these latency differences could not be explained by stereoacuity, pseudoscopic effect latencies were (on average) significantly shorter for observers with higher (relative to lower) visual acuities. In Field Study 1, binocular visual acuity predicted 9% of the variance in this latency data. Prediction improved to 18% of the variance in Field Study 2, when the monocular visual acuities of the two eyes were measured separately.

#### Why might pseudoscopic effect latency vary as a function of visual acuity?

##### Possibility #1. Observers with poor visual acuity could not see well enough

This explanation appears unlikely for several reasons. First, 85% (Field Study 1) and 89% (Field Study 2) of our observers had visual acuities that were equal to or better than 20/25. Second, even the worst binocular visual acuities recorded were deemed adequate for driving an automobile (e.g., Gresset and Meyer, 1994). Only one observer in Field Study 1 had a binocular visual acuity of 20/50 and only one in Field Study 2 had a visual acuity worse than 20/50 (she had 20/70 in one eye and 20/30 in the other). Third, observers with the worst visual acuity (including the two mentioned earlier) were removed from our regression and correlation analyses (these only examined the latencies of reversers). Fourth, several of our lower acuity reversers (e.g., 20/40 both eyes) reported the co-occurrence of four (out of a possible five) identified pseudoscopic effects. Fifth, observers can still perceive robust pseudoscopic effects with artificially blurred stereophotos, such as the scene depicted in Figure 12 (right).

##### Possibility #2. Defocus blur

Retinal images of 3D scenes contain variations in spatial blur (i.e., gradients of blur), due to the finite depth-of-focus of the eye. As a result an object will become increasingly blurred as it moves away from the focal plane (Mather, 1997; Mather & Smith, 2002; Pentland, 1987). If different parts of the scene contain sharply focused and blurred textures, then these *focused* and *blurred* regions are often perceived as being at different depths (even in the absence of other depth cues—Held, Cooper, & Banks, 2012; Nguyen, Howard, & Allison, 2005). On its own defocus blur does not indicate whether the out-of-focus object is nearer or more distant than the fixated object (only their depth magnitude). Thus, the unsigned depth information provided by defocus blur could in principle reinforce the signed depth magnitude information provided by (reversed) disparities during pseudoscopic viewing.^9^ Since defocus blur should be a more reliable depth cue for those with better visual acuity, this might explain why such observers also experienced pseudoscopic reversals more quickly and powerfully. The major problem with this explanation is that the human eye is hyperfocal at distances of 2 to 3 m (Campbell, 1957). As defocus blur is naturally more effective at near distances (not far), this particular explanation of the current results seems unlikely (since compelling pseudoscopic effects were observed here at much larger distances as far away as ∼75 m away).

##### Possibility #3. Differential binocular blur

Differential binocular blur is often reported to be more disruptive to stereoacuity than equal blur (e.g., Halpern & Blake, 1988; Westheimer & McKee, 1980). So another possible explanation of the current findings was that observers whose eyes had different visual acuities might have taken longer to experience pseudoscopic phenomena (compared to those who had similar visual acuities in their two eyes). Field Study 2 tested this possibility. However, contrary to this explanation, individual differences in the visual acuities of the two eyes were not found to significantly predict pseudoscopic effect latencies.

##### Possibility #4. Pickup of high spatial frequency content

Superior visual acuity would also have aided in the monocular pickup of higher spatial frequency content (including monocularly available texture gradient based depth information). Thus, another possible explanation of the current results was that if observers with higher visual acuity were more sensitive to monocular texture gradient cues, they should also be less prone to experience pseudoscopic phenomena (such as apparent scene depth reversals). However, the opposite pattern of results was consistently found in both of our field studies.

##### Possibility #5. Detecting disparity gradients and the consequences of pseudoscopic viewing

Superior visual acuity should also have aided in the binocular pickup of higher spatial frequency disparity content (e.g., disparity gradient-based information; see Felton, Richards, & Smith, 1972; Heckmann and Schor, 1989; Tyler, 1973, 1975), particularly that from the more distant parts of the scene. This additional disparity-based information might have reinforced stereoscopic (versus monocular) depth percepts, thereby strengthening both pseudoscopic *depth reversal* and *border reversal* effects. Those with higher visual acuity would also have been more sensitive to some of the side effects of pseudoscopic viewing, including mismatches between monocular and stereoscopic texture gradient information and the presence of invalid monocular regions (the latter might have increased the likelihood of *complex illusory occluding surfaces* being generated). According to this explanation, observers with worse visual acuity might have been restricted to detecting mismatches and side effects of pseudoscopic viewing (if at all) at object boundaries, or only between objects greatly separated in depth.

## Conclusions

In 1898, Stratton argued that the pseudoscopic viewing of natural scenes could be used to determine the limiting range of stereopsis. His technique assumed that pseudoscopic effects were not only robust and repeatable but could also be rapidly experienced. However, here we report marked individual differences in both the timing and the nature of tele-pseudoscopic phenomena. If our findings using the Pope pseudoscope are generalizable to other pseudoscopic viewing situations, then this would suggest that Stratton’s (1898) proposed method for determining the limiting range of stereopsis maybe impractical/unworkable (at least for many stereoscopic observers). However, it is possible that pseudoscopic latencies, the range of subjective experiences, and even the proportions of reversers might be quite different under different pseudoscopic viewing conditions—e.g., if the pseudoscopic viewing was conducted with a normal, as opposed to exaggerated, interocular separation.

Intriguingly, our field studies using tele-pseudostereopsis revealed complex perceptual interactions between binocular and monocular scene processing. These interactions manifested here as five discrete and identifiable categories of pseudoscopic phenomena: *apparent scene depth reversals*, *apparent object shape reversals*, *apparent size and flatness changes*, and *apparent reversals in border ownership* and even the experience of *complex illusory foreground surfaces*. The latency for experiencing pseudoscopic effects was found repeatedly to vary significantly with visual acuity. We proposed that this relationship might have been due to higher visual acuities assisting the pickup of disparity and disparity gradient information, as well as the monocular processing of higher spatial frequencies and texture gradients.

The Pope pseudoscope we used not only reversed and exaggerated available binocular disparities but also altered the direction and magnitude of the vergence eye movements required to fixate different parts of the scene. Because we did not control for, or measure, eye movements, we cannot distinguish between vergence and disparity based causes of the current pseudoscopic effects. However, we suspect that the role of reversed stereopsis may have been more important in our two field studies (as vergence appears to contribute little to distance estimates beyond about 2 to 3 m, e.g., von Hofsten, 1976).

It is worth noting that in addition to conflicts between the (exaggerated + reversed/altered) binocular and (natural) monocular depth/distance information in the current field studies, cognitive factors (such as prior knowledge and scene expectations) were also likely to have competed with the perception of the improbable shapes and depth relations generated by the pseudoscopic viewing. This interplay and competition between conflicting perceptual and top-down cognitive factors would undoubtedly be an interesting topic for future pseudoscopic research.

## Notes

1 The calculation of this 3.4 enhancement factor assumes that the observer has an actual interocular separation of 6.5 cm. However, interocular separation can vary quite markedly across individuals (e.g. Dodgson, 2004). This should lead to differences in the degree of pseudoscopic enhancement (e.g., interocular separations of 5.5 cm and 7 cm produce pseudoscopic enhancement factors of 3.6 and 3.3, respectively), which could conceivably have generated some differences in the salience and effectiveness of the pseudoscopic phenomena under investigation here.

2 While the scene looked normal for one observer when it was viewed pseudoscopically, she realized its spatial layout had been reversed when she subsequently viewed it normally. We checked for such experiences after each timing trial.

3 These complex illusory surfaces were not *illusory* in the traditional sense—as they were not *invisible foreground occluders* and their bounding contours typically had luminance/color changes across them.

4 These two nonparametric correlations are robust to the influence of outliers. The contribution of each data point is limited simply to the value of its rank and ties are corrected for.

5 One reviewer noted that unlike the other pseudoscopic experience categories, *apparent size and flatness changes* actually consisted of two perceptual phenomena (rather than a single phenomenon). Our justification for combining these phenomena together into a single category was based on our observation in Field Study 1 that they always co-occurred. In Field Study 2, of the 48 observers who reported *apparent flatness changes*, all but one of them also clearly reported *apparent size changes*.

6 If one assumed that *depth reversal* effects were responsible, then these apparent *changes in size and flatness* could potentially be explained by size-distance invariance (e.g., Epstein, Park & Casey, 1961). This would predict that to account for differences in retinal image sizes across the scene: (a) far objects (appearing near) should look smaller and flatter and (b) near objects (appearing far) should look larger and more curved. While reports consistent with prediction (a) were frequent, there were fewer reports consistent with prediction (b). Another explanation for objects appearing (on average) to be flatter during pseudoscopic viewing might be a discounting of disparity-based information relative to monocular depth/distance information.

7 Wheatstone (1852) noted that *apparent object shape reversals* were unlikely when the objects in question were both familiar and well specified by monocularly available information. Indeed, *apparent object shape reversals* appear to be particularly difficult (some claim impossible) to see for faces (Matthews, Hill, & Palmisano, 2011, 2012; Van den Enden & Spekreijse, 1989).

8 The phantom surfaces in the Häkkinen and Nyman (2001) study were not invisible foreground occluders. They were at least a superficially similar to the complex illusory surfaces reported during our natural scene field studies (both types of illusory foreground surface were textured by a mixture of empty space and visible scene details).

9 This unsigned depth information from defocus blur should also in principle reinforce perceptions of depth from binocular disparity under normal viewing conditions, as well as depth from monocular sources of depth information.
